# Differences Between Black and White Caregivers in the Association Between Autism Diagnostic Process Satisfaction and Service Use

**DOI:** 10.1007/s10803-023-06233-x

**Published:** 2024-01-17

**Authors:** Allison P. Fisher, James D. Lynch

**Affiliations:** 1 Department of Psychology, University of Cincinnati, 45 W. Corry Blvd, Cincinnati, OH USA; 2 Division of Developmental and Behavioral Pediatrics, Cincinnati Children’s Hospital Medical Center, 3333 Burnet Ave, Cincinnati, OH USA

**Keywords:** Autism, Race, Health Disparities, Service Utilization, Children

## Abstract

**Background:**

Black families of children with autism spectrum disorder have less access to high quality, culturally responsive care for their children.

**Method:**

We assessed satisfaction and service utilization among fifty (29%) Black caregivers and 124 (71%) White caregivers of children with autism spectrum disorder. We also examined whether race moderated the association between satisfaction and service utilization.

**Results:**

We did not identify racial differences in satisfaction or service utilization. Race moderated the association between satisfaction and total service use, *F*(170) = 5.29, *p* =.02, therapy service use, *F*(163) = 3.59, *p* =.046, and community service use, *F*(169) = 4.76, *p* =.046. For Black families, there was a positive association between satisfaction and service use. There was no association between satisfaction and service use among White families.

**Discussion:**

Satisfaction may be particularly important among Black families, who have been mistreated by the healthcare system and frequently face discrimination. Our results suggest the importance of culturally responsive care for Black families.

## Introduction

The autism spectrum disorder prevalence has increased in recent years, with most recent estimates indicating 1 in 36 children have received the diagnosis (Maenner et al., 2023). Thus, there is a growing population of children and families with a need for services and support. The number and types of services needed by families vary considerably based on the child’s age and clinical presentation, but the American Academy of Pediatrics recommends developmental, behavioral, and/or educational services to support children in social skills, restricted and repetitive behaviors and any other areas of need associated with the autism spectrum disorder diagnosis (e.g., language, emotion regulation). The organization recommends that services involve the family and begin as early as possible (Hyman et al., 2020; Zwaigenbaum et al., 2015). Further, management of co-occurring medical and/or behavioral conditions is also a crucial part of comprehensive autism spectrum disorder intervention (Hyman et al., 2020).

Given the substantial empirical support for autism spectrum disorder interventions (Fuller & Kaiser, 2020; Sandbank et al., 2020; Smith & Iadarola, 2015), it is important to maximize access to these services for families. Critical to this goal is identifying what factors may limit service access and how different factors may interact to exacerbate or ameliorate barriers to access. There are notable disparities in service access and utilization for minoritized and low-income families of children with autism spectrum disorder across specialized care, educational, and community services (Bilaver & Havlicek, 2019; Smith et al., 2020). Black/African American families are particularly vulnerable to experiencing health care inequities due to the pervasive history of systemic and institutional racism faced by this population. A large body of research has highlighted differences in the number and quality of services received by Black families of children with autism spectrum disorder compared to White families of children with autism spectrum disorder. Black families of children with autism spectrum disorder enter treatment at later ages (Yingling & Bell, 2019) and have lower rates of outpatient and specialty service use than White families (Bilaver et al., 2021; Broder-Fingert et al., 2013; Kalb et al., 2012). Additionally, systemic barriers limit utilization of services, such as geographic accessibility of services, differences in service quality between public and private insurance, and limited diversity of intervention research (Colic et al., 2020).

Black families also face disparities in the quality of care they receive, particularly when it comes to culturally responsive care. Black families of children with autism spectrum disorder have reported distrust of receiving equal clinical healthcare (Burkett et al., 2015), lower rates of family-centered care (Magaña et al., 2015; Montes & Halterman, 2011), and experiences with bias and racism from providers (Burkett et al., 2015; Dababnah et al., 2018; Weitlauf et al., 2023). Black families are more likely to report that doctors do not spend enough time with their child than White families (Magaña et al., 2015). Additionally, Black parents of children with autism spectrum disorder have reported negative experiences with the diagnostic process due to institutionalized barriers, communication differences, and discrimination (for a review, see Fisher et al., 2022). Providers themselves have reported disparities in care for Black families, including being less comfortable identifying early symptoms of autism spectrum disorder in Black children and believing that Black parents are less knowledgeable about the diagnosis than White parents (Zuckerman et al., 2013).

Importantly, a lack of culturally responsive care and dissatisfaction with received services can negatively impact future therapeutic service use. Personally mediated racism (i.e., racism directed towards and/or experienced by an individual) impacts healthcare utilization, provider-family interactions, and trust in healthcare providers (Bleich et al., 2019; Maina et al., 2018; Paine et al., 2018). One study described that over one-fifth of Black adults avoided seeking healthcare for themselves and for family members due to anticipated discrimination (Bleich et al., 2019). Additionally, Maina et al. (2018) found that greater implicit bias among providers was associated with lower ratings of patient-centered care. Experiences of discrimination also contribute to medical mistrust (Williamson et al., 2019), which is an important predictor of healthcare utilization (Arnett et al., 2016).

For families of children with autism spectrum disorder, the diagnostic process is the gateway to specialized healthcare services as it is typically the point at which children are formally diagnosed. The diagnosis can then act as a “key” that opens the doors to specialized therapeutic services and resources. Importantly, the diagnostic process can vary considerably across families. For some families, the child may be diagnosed in a single visit by their primary care provider. For others, the process may involve a multi-disciplinary team over several visits (e.g., primary care, developmental pediatricians, developmental psychologists, speech therapists, school psychologists). Further, the diagnostic process involves the *manner* in which the diagnosis and other evaluation findings are shared with families. An important part of the process is the way in which providers guide families towards next steps, including recommendations and referrals. Given the critical role the diagnostic process has in opening the door to subsequent specialized healthcare services, it reasons that patients who are more satisfied with that process would be more likely to engage with recommended/referred services. In fact, Parish et al. (2012) found that satisfaction with their child’s care drove service utilization among Latino caregivers of children with autism spectrum disorder; however, the types of clinical encounters were not specified. Moreover, this association has not been examined among African American families. Thus, the present study aims to address the dearth of research in this area by examining associations between families’ satisfaction with the process of receiving an autism spectrum disorder diagnosis for their child and their subsequent service utilization. First, we hypothesize that Black families will report less satisfaction with the diagnostic process of autism spectrum disorder than White families. We also hypothesize that Black families will use services at lower rates than White families. Finally, we hypothesize that race will moderate the association between satisfaction and service utilization, such that satisfaction with the diagnostic process will have a stronger positive association with service utilization for Black versus White caregivers.

## Method

### Setting

At a large academic medical center in the Midwest, young children with developmental concerns and possible autism spectrum disorder are referred for an evaluation in one of several models. Children under 3 are assessed by a developmental pediatrician, speech-language pathologist, and clinical psychologist. Children ages 3 to 5 are evaluated by a psychologist and speech-language pathologist. Children aged 6 and older are evaluated by a psychologist alone. Evaluators provide detailed reports with findings and recommendations, such as referrals for school-based evaluations, speech therapy, and behavior therapy. A developmental behavioral pediatrician or psychologist discusses evaluation results and relevant resources with the family in a feedback session.

### Participants

We obtained contact information for all children who were diagnosed with autism spectrum disorder in 2018 through the medical center from the medical record and attempted to contact each participant via phone, email, and text from February 2021 to February 2023. We chose the year 2018 to examine both the diagnostic process and subsequent service utilization in the two years following diagnosis prior to the COVID-19 pandemic, which significantly impacted evaluations and treatment service provision. We also wanted to allow families to have time to engage in services, particularly given long waiting lists for specialized services (Yingling & Bell, 2019b). We consulted with a local non-profit organization for Black and Brown caregivers of a child with autism spectrum disorder who believed that caregivers would be able to remember and recall details from the diagnostic process in 2018, given its significance and salience to families.

Participants were eligible to participate in the study if they identified as a White/Caucasian or Black/African American caregiver of a child diagnosed with autism spectrum disorder through Cincinnati Children’s Hospital Medical Center. We also included bi-racial/multi-racial caregivers, when at least one race was described as Black, African, or African American. We included bi-racial and multi-racial caregivers because they are also exposed to anti-Black institutionalized and personally mediated racism (Forrester et al., 2019; Franco et al., 2021; Singh et al., 2017). Caregivers needed to speak one of the following languages to participate: English, Spanish, Nepali, Arabic, or French. However, all participants in the current study spoke English fluently. Please note, we will be using the term “Black” to describe Black and African American participants across studies to be as inclusive as possible.

### Recruitment

This study was approved by the Cincinnati Children’s Hospital Medical Center Institutional Review Board. A racially and ethnically diverse team of multi-disciplinary researchers (i.e., developmental behavioral pediatricians, psychology trainees, speech-language pathology trainees, and family members of children with Autism) recruited families by phone and email identified through patients’ electronic health records. In recruitment calls, we emphasized that we wanted to hear families’ voices to be able to improve care for families moving forward. We made efforts to highlight that nothing families shared would impact them or their child’s care. Approximately halfway through recruitment, the research team created a 90 second video over zoom introducing families to the study to increase rapport and trust. The video was sent to families via email and text. The aim of the video was to allow families to be able to see the researchers conducting recruitment and highlight the team’s multi-disciplinary nature and investment in the research project to improve the quality of care families’ receive.

### Chart Review Variables

Variables extracted from the electronic health record included the arena model type, IQ or developmental score, Vineland score, family address, caregiver language, days between the evaluation and the feedback, child’s age at the time of the evaluation, child’s sex, child’s diagnoses, and public insurance status (yes/no). We also extracted the number and type of recommendations in the evaluation reports (e.g., recommendation for speech therapy). Cognitive z-scores were derived by taking an individual patient’s cognitive score from their evaluation report and calculating a z-score using the normed population mean and standard deviation from the specific standardized assessment measure that was used in the evaluation. This was done to standardize scores across all patients for analyses, regardless of the cognitive score type (i.e., T-scores, standard scores).

### Census Tract Data

We extracted the 2018 median household income and the Childhood Opportunity Index 2.0 (COI) from the Census Tract, which is a geographic area of approximately 4,000 individuals by zip code.

#### Childhood Opportunity Index

The COI measures and maps the quality of resources and conditions in a child’s neighborhood at the census tract level. Domains include measures of the quality of education (e.g., early childhood education centers, third grade reading and math proficiency, teachers’ years of experience), health and environment (e.g., access to healthy food, house vacancy rate, hazardous waste dump sites), and social and economic neighborhood resources and conditions (e.g., homeownership rate, median household income, single-parent household; Noelke et al., 2020). COI scores are percentiles compared to national norms, where lower scores reflect worse childhood opportunities in the census tract.

### Surveys

Survey responses were collected via REDCap, a secure online survey and questionnaire database. Caregivers were given the option of completing the surveys by verbally providing their responses over the phone to a member of the research team, or by completing the surveys at a later time online via a secure link sent by email.

### Demographic Information

We asked caregivers their race, ethnicity and gender over the phone. Caregivers completed additional demographic questions, providing their age, relationship to the child, years of education (on a scale from less than 12 to greater than 16), employment status, marital status, and household income.

### Service Receipt

Families selected what outpatient and/or private services they or their child accessed since receiving the autism spectrum disorder diagnosis from a list of services typically recommended in evaluation reports (e.g., speech therapy, behavior therapy, caregiver support group, Applied Behavior Analysis). Caregivers were able to describe any additional services they or their child received that were not provided as a response option.

### Satisfaction

The satisfaction survey was developed through an iterative process. The researcher created a team comprised of two White psychology graduate students, a White licensed psychologist, a White developmental behavioral pediatrician fellow, a White speech pathology graduate student, a White epidemiologist, and a Black family member of a child with autism spectrum disorder. The team conducted a systematic review of caregivers of color’s experiences with the diagnostic process. Based on the literature, the team drafted questions to assess caregivers’ satisfaction with the diagnostic process within the clinic, which was measured on a 7-point Likert scale from “Strongly Agree” (1) to “Strongly Disagree” (7), representing the extent to which they agreed that they were satisfied with different aspects of care. Questions were categorized into four domains: *provider factors* (e.g., “I felt emotionally supported by my providers”), *information provided to families* (e.g., “I was satisfied with the recommendations, resources, and referrals I received”), *wait times* (e.g., “I was satisfied with the amount of time between my child’s first evaluation appointment and receiving the official diagnosis at the Information Sharing Session [ISS]”), and *“other”* (i.e., “My providers respected my cultural and family values”; “I was satisfied with the written results [the reports]”). The team continuously updated the survey in response to feedback from (1) two developmental behavioral pediatricians of color, (2) members of a non-profit organization for Black and Brown caregivers (all of whom were caregivers themselves), and (3) the family advisors for Cincinnati Children’s Hospital Medical Center, who are family members of children with disabilities including autism spectrum disorder, who received care at Cincinnati Children’s Hospital Medical Center. We conducted a multigroup confirmatory factor analysis (MG-CFA) to test measurement invariance in the satisfaction survey across Black versus White participants (Sass et al., 2014) We identified high factor loadings (0.56-0.97) and evidence for measurement invariance (Change in Comparative Fit Index [CFI] < 0.01). Though acceptable for White participants, the Root Mean Squared Error of Approximation (0.20) and CFI (0.81) for Black participants were slightly below the “acceptable” range.

### Data Analysis

#### Demographic Variables

We examined means and percentages of demographic variables, satisfaction survey responses, and service utilization. Using independent samples t-tests, we examined whether demographic characteristics or service utilization differed between Black and White families. We also examined differences in demographic characteristics for families who participated in the study in comparison to those who did not. We reported effect sizes using Cohen’s D.

### Control Variables

We selected potential control variables based on their theoretical significance to the diagnostic process. For example, families may be less satisfied if their child was diagnosed at an older age. Control variables in all analyses included the caregivers’ job status and the child’s co-occurring conditions (i.e., genetic or medical condition, language disorder, emotional or behavioral disorder), IQ, age of evaluation, and sex. In several large-scale studies, segregation, income, education, and neighborhood factors have been used as a proxy for institutionalized racism and may be more robust predictors of adverse health outcomes than health risk behaviors (e.g., diet, exercise, alcohol consumption) for Black adults (Simons et al., 2018, 2021). Therefore, we included several control variables as a proxy for institutionalized racism in all analysis models, including caregivers’ years of education, public insurance status, household income, and caregivers’ COI scores.

### Statistical Models

Statistical analyses were conducted using PROCESS (Hayes, 2017) in SPSS Version 28.0 (SPSS, 2021).

#### Aim 1: Does Race Predict Satisfaction with the Diagnostic Process?

To address Aim 1, we conducted separate unadjusted generalized linear models (GLM) examining the relation between race and the satisfaction total score and race and satisfaction domain scores. Next, we conducted adjusted models with the abovementioned control variables.

#### Aim 2: Does Caregiver Race Predict Entry into Services?

To address Aim 2, we conducted separate unadjusted GLMs examining the relation between race and the proportion of recommended services received (e.g., family received 60% of services recommended in evaluation reports) and by category (i.e., outpatient therapies, community services). For Aims 1 and 2, we used backward stepwise regression to eliminate non-significant variables (*p* >.10).

#### Aim 3: Does Race Moderate the Association Between Satisfaction and Service Utilization?

To test whether the association between satisfaction with the diagnostic process and service utilization is moderated by race, we ran a linear regression in which service utilization was regressed onto satisfaction domain scores, race, and an interaction term (race x satisfaction). We examined service utilization by the proportion of recommended services received total and by category. In this aim, we controlled for median income, as it was significantly associated with service utilization. In all models, we used the false discovery rate (FDR) to adjust for multiple comparisons and maintain power. Results are reported using FDR-corrected *p*-values.

## Results

### Background Characteristics

Of the 389 children who were Black or White and were evaluated for autism in 2018, 50 (29%) Black caregivers and 124 (71%) White caregivers completed the satisfaction survey (total sample = 174; 44.7% of all children evaluated). The time from diagnostic evaluation to study participation was approximately 4 years (*SD* = 0.42 years, *Range* = 2.5 to 5.1).

### Survey Completers and Non-completers

Nineteen caregivers (11%) completed the survey by phone, with no differences in the mean satisfaction between those who completed the survey by phone in comparison to those who completed it online (*t*(173) = 1.46, *p* =.15). Forty-five (11.8%) participants agreed to participate but did not complete the survey after several reminders; 114 (29.8%) did not respond to multiple call and or email attempts (passive decline); 13 (3.4%) declined to participate over the phone or email; 32 (8.4%) did not have an active phone number (number no longer in service) or email address on file. Caregivers of Black children (*n* = 59, 62.8%) were more likely than caregivers of White children (*n* = 118, 41.1%) to participate. Caregivers of White children were more likely to passively decline (*n* = 105, 36.6%) than caregivers of Black children (*n* = 9, 9.6%).

Comparing completers vs. non-completers with Black children, there were no differences in demographic, socioeconomic, evaluation (e.g., cognitive, adaptive scores), or pre-evaluation service utilization characteristics. Comparing completers vs. non-completers with White children, caregivers who completed the survey had higher median incomes (Completers: *Mdn* = $87,477, *SD* =$32,035; Non-completers: *Mdn* = $75,628, *SD* = $28,112) and lower levels of vulnerability on the COI (Completers: *M* = 0.017, *SD* = 0.03; Non-completers: *M* = 0.008, *SD* = 0.03) in comparison to the median income and COI scores of non-completers (*t*(285) = 2.6–3.3, *p* =.001 −.01, Cohen’s *ds* = 0.28-0.40). Two participants did not complete cognitive or developmental testing, and one participant did not complete the Vineland. We were unable to identify the Childhood Opportunity Index or Median Income for the address of one participant. Because less than 2% of participants had missing data, we removed them from analyses that included these variables as control variables.

### Study Participants

Socio-demographic characteristics for Black and White children and caregivers are presented in Table 1. Across both races, most caregivers (*n =* 150, 86.2%) were mothers. The mean age of caregivers was 38.1 years old (*SD* = 7.2). Approximately one-quarter (*n* = 42, 24.1%) had a high school education equivalency or less. Seventy-eight caregivers held full-time jobs (44.8%), and 58 (33.3%) caregivers stayed home to care for their children. The median family income was $72,126. Children were on average 3.2 years old (*Mdn* = 3 years, *SD* = 1.6) at the time of the evaluation. The mean wait time between the evaluation and feedback was 49 days (*SD* = 64; *Mdn* = 27 days), skewed higher due to large outliers, which likely represent families who needed to reschedule their feedback multiple times.

**Table 1:** Sample demographic characteristics by race

	Black (*n* = 50)	White (*n* = 124)	Cohen’s d/Phi^a^	p
**Child demographics**				
Child gender, *n* (%) Male Female	42 (84%) 8 (16.0%)	97 (78.2%) 27 (21.8%)	0.07	0.39
**Caregiver demographics**				
Relationship to child, *n* (%)				
Mother Father Adoptive parent Grandmother Legal guardian	46 (92.0%) 4 (8.0%) 0 (0.0%) 0 (0.0%) 0 (0.0%)	104 (83.7%) 10 (8.1%) 4 (3.2%) 3 (2.4%) 3 (2.4%)	0.16	0.64
Age, *M (SD)*	36.40 (6.88)	38.32 (8.41)	-0.33	0.06
Gender, *n* (%)			0.049	
Female Male	46 (92.0%) 4 (8.0%)	110 (88.7) 14 (11.3%)		0.51
Relationship status, *n* (%)			0.10	
Single Married/living with a partner In a committed relationship Divorced Other	23 (46.0%) 14 (28.0%) 7 (14.0%) 6 (12.0%) 0 (0.0%)	12 (9.7%) 94 (75.8%) 10 (8.1%) 6 (4.8%) 2 (1.6%)		< 0.001
Education, *n* (%)			0.087	
High school degree or less Some college Bachelor’s degree Advanced degree	15 (30.0%) 23 (46.0%) 9 (18.0%) 3 (6.0%)	27 (21.8%) 41 (33.1%) 31 (25.0%) 25 (20.2%)		0.25
Occupation status, *n* (%)^b^			0.19	
Full-time Part-time Multiple part-time Contract Unemployed Unable to work Stay-at-home caregiver Other	21 (42.0%) 6 (12.0%) 0 (0.0%) 2 (4.0%) 3 (6.0%) 3 (6.0%) 14 (28.0%) 1 (2.0%)	57 (46.3%) 13 (10.6%) 3 (2.4%) 1 (0.8%) 4 (3.3%) 1 (0.8%) 44 (35.8%) 0 (0.0%)		0.098
Public insurance status, *n* (%)	37 (74.0%)	48 (38.7%)	-0.32	< 0.001
Median income (2018, *M [SD]*)	$50,039.9 (32,590.5)	$85,731.8 (32,590.5)	-1.12	< 0.001
COI Z-score, *M (SD)*	27.7 (27.97)	63.6 (28.6)	-1.35	< 0.001
**Pre-Evaluation Service Utilization**				
Therapies prior to evaluation, *n* (%)				
Speech	25 (50.0%)	58 (46.8%)	− 0.02	0.77
Occupational Therapy	13 (26.0%)	50 (40.3%)	0.14	0.06
Physical Therapy	7 (14.0%)	16 (12.9%)	− 0.015	0.85
Early Intervention	22 (44.0%)	61 (49.2%)	0.054	0.47
Preschool	19 (38.0%)	51 (41.1%)	0.021	0.77
Individualized education plan	16 (32.0%)	39 (31.5%)	− 0.005	0.94
**Evaluation Characteristics**				
Age at evaluation (months, *M [SD]*)	41.1 (17.7)	42.5 (19.7)	-0.07	0.33
Cognitive z-score, *M (SD)*	-2.3 (0.86)	-1.8 (1.3)	-0.40	0.006
Vineland Total Score, *M (SD)*	68.8 (11.0)	69.0 (9.8)	-0.07	0.69
Days between evaluation and feedback, *M (SD)*	52.8 (75.6)	50.0 (76.4)	0.07	0.71
Co-occurring diagnoses, *n* (%)^c^				
Hear	0 (0.0%)	2 (1.6%)	0.068	0.37
Delay	38 (76.0%)	79 (63.7%)	− 0.12	0.12
Behavioral or emotional diagnosis	3 (6.0%)	20 (16.1%)	0.14	0.07
Medical condition	1 (2.0%)	7 (5.6%)	0.079	0.30
Language disorder	15 (30.0%)	40 (32.3%)	0.022	0.77

^a^Cohen’s D used for continuous variables and Phi used for categorical variables

^b^one caregiver did not disclose employment status

^c^Hear = hearing diagnosis, such as mild conductive hearing loss; Delay = Developmental delay in one or more areas; Behavior or emotional diagnosis, such as Attention-Deficit/Hyperactivity Disorder, Adjustment disorder, disruptive behavior disorder; Medical conditions such as Spina Bifida, seizures, Fragile X syndrome; Language disorder such as mixed-receptive expressive language disorder

#### Aim 1: Does Race Predict Satisfaction with the Diagnostic Process?

In unadjusted models, there were no differences between Black and White responses regarding the total satisfaction score (*B* = 0.16, *p* =.37), provider factors (*B =* 0.001, *p* =.99), information provided (*B* = 0.33, *p* =.17), or wait times (*B =* 0.24, *p* =.32). In fully adjusted models, race did not predict satisfaction with the diagnostic process. On average, satisfaction domain and total scores ranged between 1.97 and 2.49, corresponding to “agreement” with the statements on the questionnaire (See Table 2).

**Table 2:** Satisfaction scores by question and race

Satisfaction scores ^a^	Black, M (SD)	White, M (SD)
**Provider factors**		
The providers explained the diagnostic process.	1.72 (0.83)	1.99 (1.31)
I felt understood by the DDBP providers when I talked about my child.	1.98 (1.38)	1.90 (1.20)
The DDBP providers respected and valued my opinions and point of view.	1.89 (1.20)	1.85 (1.11)
I felt emotionally supported by my providers.	2.42 (1.75)	2.37 (1.55)
I had the opportunity to ask questions about the diagnosis.	1.82 (1.04)	1.73 (0.99)
**Provider total**	1.97 (0.99)	1.97 (0.97)
**Information provided**		
I was satisfied with the recommendations, resources, and referrals I received.	2.18 (1.37)	2.57 (1.69)
I was satisfied with the amount of information I received about my child’s diagnosis and potential next steps.	2.08 (1.38)	2.51 (1.68)
My providers gave me enough guidance and resources to begin to understand my child’s diagnosis.	2.12 (1.27)	2.38 (1.59)
My providers gave me enough guidance and resources to get me started on next steps (e.g., school, therapy).	2.25 (1.56)	2.48 (1.62)
**Information total**	2.16 (1.25)	2.49 (1.50)
**Wait times**		
I was satisfied with the amount of time between the referral to DDBP and my child’s first Autism evaluation appointment.	2.32 (1.62)	2.75 (1.78)
I was satisfied with the amount of time between my child’s first evaluation appointment and receiving the official diagnosis at the Information Sharing Session (ISS).	2.26 (1.60)	2.32 (1.54)
**Wait total**	2.16 (1.25)	2.54 (1.46)
**Other**		
My providers respected my cultural and family values.	1.84 (0.82)	1.71 (0.94)
I was satisfied with the written results (the reports).	2.18 (1.12)	2.12 (1.23)

^a^ Survey items were rated on a 7-point Likert scale (1 = Strongly agree to 7 = Strongly disagree)

#### Aim 2: Does Caregiver Race Predict Entry into Services Post-evaluation?

In unadjusted models, total service use (*B* = 0.04, *p* =.30), therapeutic services received (*B* = 0.083, *p* =.08), community resources received (*B*=-0.12, *p* =.80) did not differ by race. In fully adjusted models, race did not predict total services used or the number of therapy or community resources accessed. See Table 3 for recommended and received services by race.

**Table 3:** Therapeutic services received following the evaluation

Service	Black (*n =* 50)	White (*n =* 124)	Effect Size	p-value
**Therapies, N (%)**				
*Speech*				
Recommended (yes)	34 (60.8%)	75 (60.5%)	0.16	0.36
Received if recommended (yes)	29 (85.0%)	64 (85.3%)	− 0.001	0.96
*Occupational Therapy*				
Recommended (yes)	17 (34.0%)	46 (37.1%)	− 0.064	0.35
Received if recommended (yes)	16 (94.0%)	41 (89.1%)	0.17	0.70
*Physical Therapy*				
Recommended (yes)	1 (2.0%)	2 (1.6%)	-	-
Received if recommended (yes)	1 (100.0%)	2 (100%)	-	-
*Behavior Therapy*				
Recommended (yes)	21 (42.0%)	76 (61.3%)	− 0.39	0.02
Received if recommended (yes)	3 (14.0%)	33 (43.4%)	− 0.62	0.014
*Group Therapy*				
Recommended (yes)	22 (44.0%)	65 (52.4%)	− 0.17	0.32
Received if recommended (yes)	0 (0.0%)	12 (18.5%)	− 0.54	0.03
*ABA Services*				
Recommended (yes)	40 (80.0%)	99 (79.8%)	0.004	0.91
Received if recommended (yes)	6 (15.0%)	27 (27.3%)	− 0.29	0.13
*Early Intervention*				
Recommended (yes)	10 (20.0%)	24 (19.4%)	0.016	0.92
Received if recommended (yes)	7 (70.0%)	16 (66.7%)	0.069	0.86
***Total number of therapeutic services recommended***	3.56 (1.67)	3.81 (1.71)	− 0.15	0.39
**Community resources**				
*Ohio Autism Scholarship*				
Recommended (yes)	32 (64.0%)	75 (60.4%)	0.072	0.67
Received if recommended (yes)	2 (6.0%)	30 (32.2%)	− 0.29	0.17
*Board of Developmental Disabilities*				
Recommended (yes)	40 (80.0%)	98 (79.0%)	0.024	0.89
Received if recommended (yes)	20 (50.0%)	42 (42.8%)	0.14	0.45
*Connect with social work*				
Recommended (yes)	49 (98.0%)	121 (97.5%)	0.028	0.87
Received if recommended (yes)	12 (24.4%)	23 (19.0%)	0.14	0.43
*Connect with family Support Professionals*				
Recommended (yes)	19 (38.0%)	50 (40.3%)	− 0.047	0.78
Received if recommended (yes)	4 (21.0%)	10 (20.0%)	0.026	0.92
Total number of community resources recommended	3.26 (1.19)	3.21 (1.24)	0.088	0.60

Note. Families may not have been recommended a service if they were already receiving that service at the time of the evaluation. We did not examine differences in physical therapy use by race given the small number of children participating in this therapy

#### Aim 3: Does Race Moderate the Association between Satisfaction and Service Utilization?

**Fig. 1:**
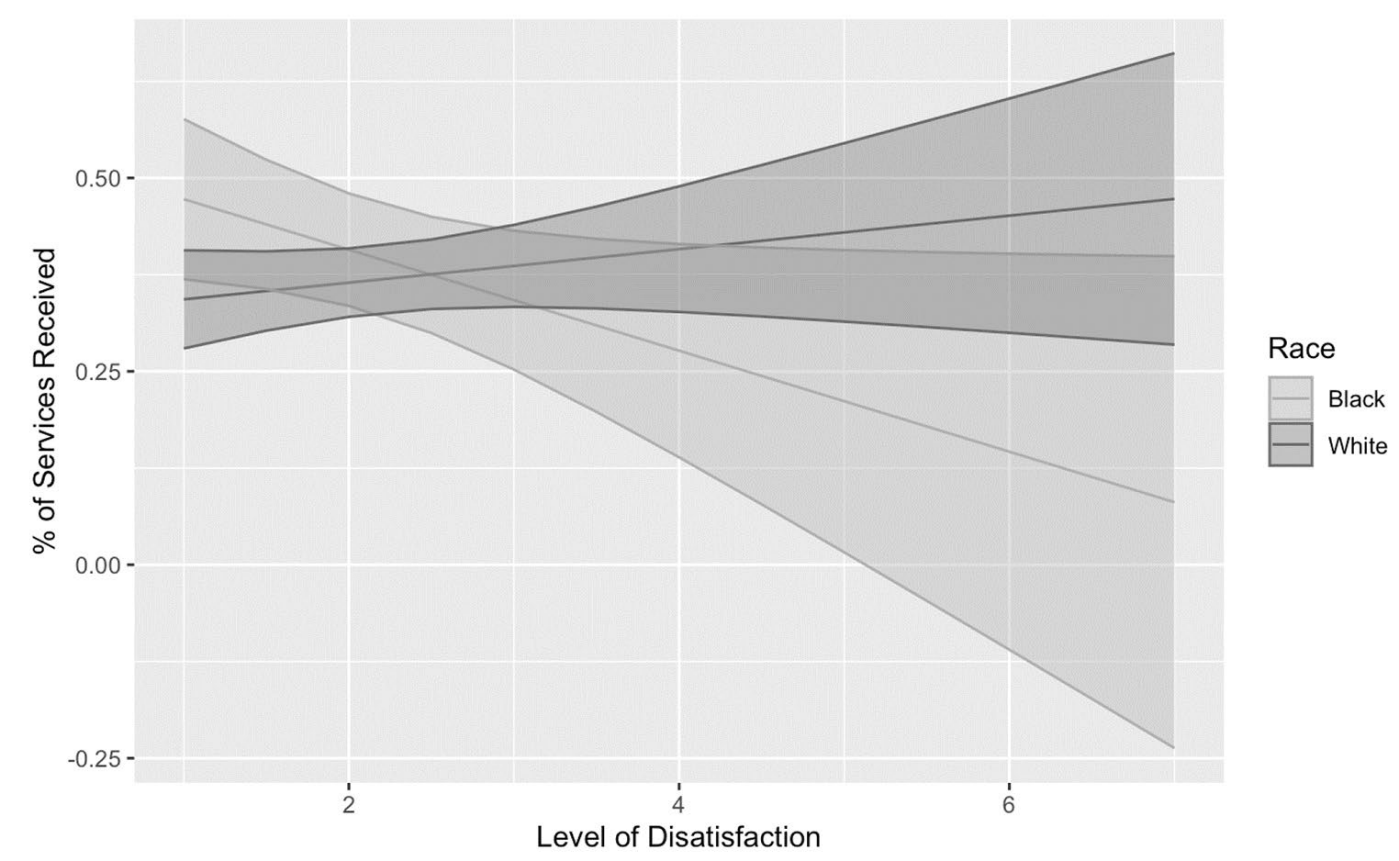
Race as a moderator of satisfaction on total service utilization. Note. Level of satisfaction: 1 = High level of satisfaction; 7 = High level of dissatisfaction

Race moderated the association between satisfaction and total service use, *F*(170) = 5.29, *p* =.02, satisfaction and therapy service use, *F*(163) = 3.59, *p* =.046, and satisfaction and community resource use, *F*(169) = 4.76, *p* =.046. When decomposing the interaction, greater satisfaction was associated with greater total service use among Black (*B* = − 0.050, *t*(170)= -2.01, *p* =.046) but not White families (*B* = 0.031, *t*(170) = 1.25, *p* =.21, Fig. 1).

**Fig. 2:**
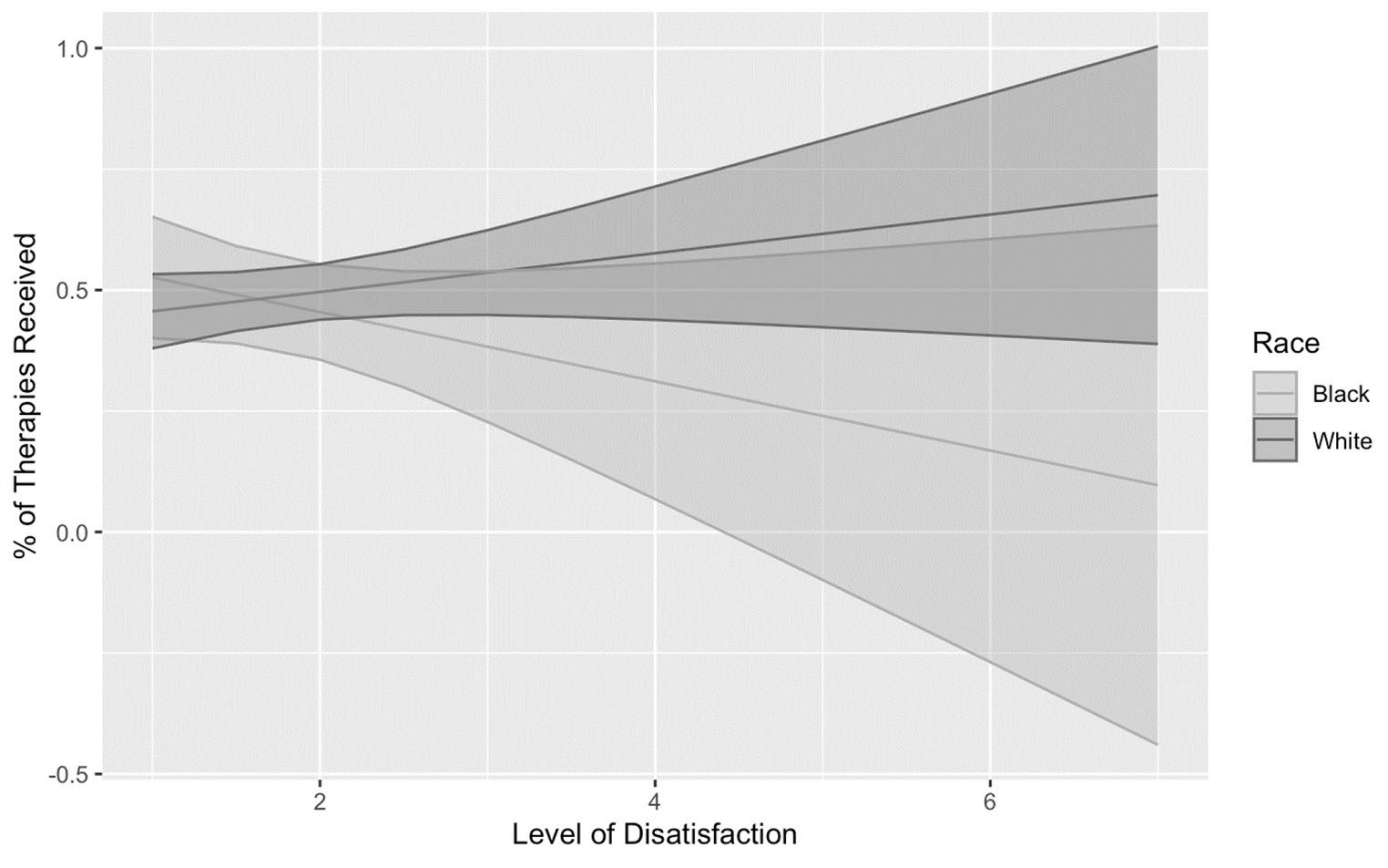
Race as a moderator of satisfaction on therapeutic service utilization. Note. 1 = High level of satisfaction; 7 = High level of dissatisfaction

Greater satisfaction was marginally associated with a greater percentage of therapeutic service utilization among Black families, *B* = − 0.061, *t*(163) = -2.56, *p* =.08. Among White families, there was no association between satisfaction and service utilization, *B* = 0.034, *t*(163) = 1.26, *p* =.21, Fig. 2.

**Fig. 3:**
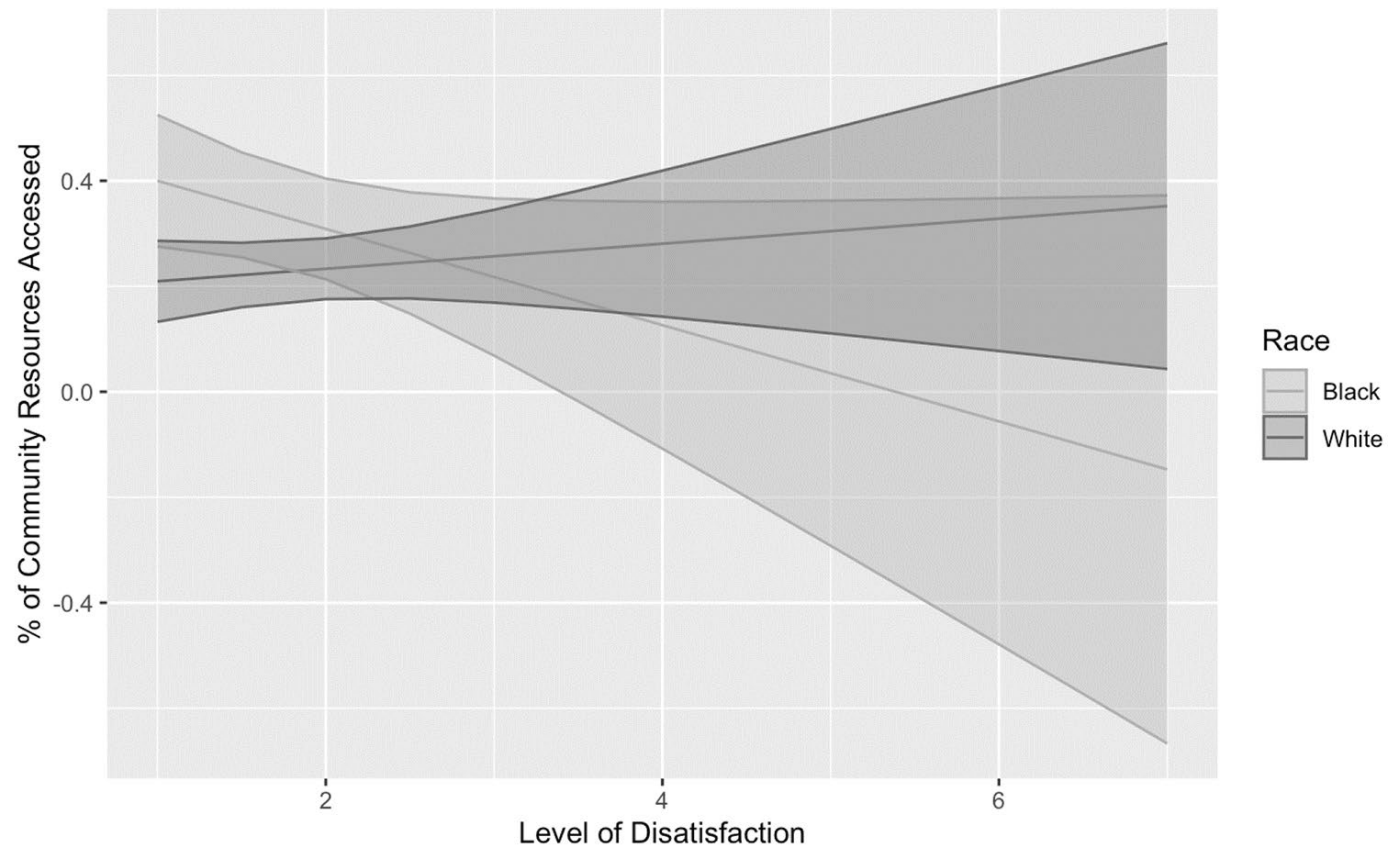
Race as a moderator of satisfaction on community resource utilization. Note. Level of satisfaction: 1 = High level of satisfaction; 7 = High level of dissatisfaction

For Black families, there was a positive association between satisfaction and community resource utilization, *B* = − 0.062, *t*(169) = -2.19, *p* =.03. There was no association between satisfaction and community resource utilization for White families, *B* = 0.021, *t*(169) = 0.97, *p* =.33, Fig. 3.

## Discussion

We found that most families were satisfied with the autism spectrum disorder diagnostic process at a large academic medical center, and there were no differences in families’ satisfaction by race. After their child was diagnosed with autism, most families reported receiving speech and occupational therapy. Most families also received school-based services at their child’s local school district. Utilization was lower for other recommended services. We identified that race moderated the associations between satisfaction and therapy use and satisfaction and community resource use, such that there was a significant positive association between satisfaction and service use for Black families.

### Satisfaction

Only two previous studies have examined the association between race or ethnicity and satisfaction with the diagnostic process. A U.S. study did not identify racial differences in satisfaction but noted minoritized families felt evaluation and recommendation needs were less met (Jashar et al., 2019). The other study was conducted in New Zealand and did not identify differences between the satisfaction of New Zealand European families compared to Māori and Pacific Islander families (Eggleston et al., 2019). In both studies, limited representation of minoritized caregivers precluded meaningful conclusions.

We were surprised to find similar levels of satisfaction between Black and White families in this study, given the vast literature suggesting Black families experience reduced quality of care and often do not receive culturally responsive care (Dababnah et al., 2018; Magaña et al., 2015; Stahmer et al., 2019; Weitlauf et al., 2023). Importantly, families may have been more satisfied with their evaluation because it was conducted by a multi-disciplinary team of providers with specialized training in diagnosing autism spectrum disorder. Further, subsequent referrals and recommendations are largely made within the same institution as the evaluation. Thus, Black and White families may have been similarly satisfied with learning that they could receive these interventions both in a setting and with providers with whom they are already familiar. Additionally, across racial groups, most children were evaluated before age 4, and early age of diagnosis has been associated with greater satisfaction in past research (Guillon et al., 2022; Sansosti et al., 2012).

### Service Utilization

We were surprised to find service utilization did not differ between Black and White families, particularly in the context of greater levels of neighborhood vulnerability and lower median income among Black families in our sample. Our findings conflict with previous research suggesting racial disparities in service utilization and systemic barriers to service use. It may be that high levels of satisfaction drove greater service use among Black families in this sample. Families were able to receive services at the diagnosing institution, potentially facilitating service use and reducing barriers to care. Relatedly, families in this sample participated in the diagnostic process and most children were evaluated before age 4, suggesting potentially fewer barriers to service use.

Despite no overall differences between Black and White families in total use of therapies or community resources, Black families were less likely to use recommended behavior or group therapy. Differences in utilization in these therapies may have been due to a combination of systemic barriers to care (Bilaver & Havlicek, 2019; Smith et al., 2020), a lack of trust in providers (Arnett et al., 2016), or stigma associated with emotional or behavioral disorders (Planey et al., 2019).

### Race Moderated the Association between Satisfaction with the Diagnostic Process and Service Utilization

We identified that race moderated the association between satisfaction with the diagnostic process and service use, such that greater satisfaction was associated with greater total service, community resource, and therapy service use among Black, but not White, families. Therefore, satisfaction may be particularly important for Black families, who have been mistreated by the healthcare system and frequently face discrimination in their everyday lives and by healthcare providers (Bey et al., 2019; Bleich et al., 2019; Maina et al., 2018). Positive healthcare experiences can facilitate trust in providers and their recommendations, particularly during an emotional and challenging time for caregivers (Abel & Efird, 2013; Haywood et al., 2014). Future studies could examine whether results are replicated with a larger sample, including other variables that may also be important to service utilization, such as trust in the recommendations, transportation, and flexibility in caregivers’ schedules (Henning-Smith et al., 2013; Pickard & Ingersoll, 2015; Stormacq et al., 2019). In a future study, we could explicitly ask caregivers if their experiences with the diagnostic process influenced their service utilization, which could provide us with greater insight into caregivers’ perspectives.

### Limitations

This study should be interpreted in the context of its limitations. Though we developed the survey through an iterative process that included participants in the diagnostic process (e.g., psychologists, speech pathologists, families), we did not engage in a formal cognitive validation, which may impact the validity and comprehensibility of the survey. We also limited the number of questions on the satisfaction survey to reduce the burden of participation. For example, we only asked one question about families’ culture: “My providers respected my cultural and family views.” Future studies could include more questions about the way in which families’ culture, perceptions of disability, and providers’ ability to deliver culturally responsive care impacted the diagnosis process.

We recruited a representative sample of Black caregivers in terms of demographic variables; however, we only recruited caregivers who were evaluated at a large academic medical institution in the Midwest. This is a major limitation impacting generalizability of our study’s results, given that the characteristics of families evaluated at other agencies, those who are not evaluated, or those who are in different geographic regions are likely to be different. Moreover, we did not recruit a representative sample of White participants. White caregivers in our study had higher median incomes and lived in less socially vulnerable neighborhoods in comparison to white caregivers who did not participate. Previous studies have suggested that greater socioeconomic status is associated with greater satisfaction with the diagnostic process and service utilization (Eggleston et al., 2019; Hidalgo et al., 2015; Morin et al., 2022; Sansosti et al., 2012); therefore, the satisfaction ratings and service use of White caregivers may be inflated in our study.

Though the study focused on the experiences of Black and White caregivers, we are missing the perspective of other racial and ethnic groups and linguistically diverse individuals. Finally, due to the small sample size, we were unable to examine differences in the experiences of families who identify as Black, bi-racial, and African. Colorism, experiences of discrimination and systematic racism against people with darker skin tones, is prevalent in everyday life and the healthcare system, which may impact families’ experiences with the diagnostic process (Slaughter-Acey et al., 2019; Stamps et al., 2022). Further, caregivers who identify as African may have different experiences due to the intersection of their nationality, skin tone, race, and lived experiences (Asante et al., 2016).

We are limited by caregivers’ retrospective reports of satisfaction and service utilization in the current study. Responses to the satisfaction survey in our study may be positively biased as families are far removed from their child’s diagnostic process and have been engaged in several services. The outcomes might be influenced by social desirability, as caregivers could be answering based on what they think is expected of them or what researchers wish to hear. Similarly, families may have conflated their experiences with the diagnostic process with more recent healthcare experiences. We are also uncertain about the accuracy of caregivers’ reports of their child’s service engagement, as caregivers may not always understand the terminology used to describe treatments. We do not know the timing, dose and intensity of services. Lastly, families with very low levels of satisfaction with services received at the medical center of the present study may have been much less willing to participate in a research study at that same institution.

## Conclusion

Together, the results from the present study suggest that at a large, well-resourced academic medical center, families have relatively high levels of satisfaction across racial groups. However, results also suggest that satisfaction with the diagnostic process is a particularly influential factor that promotes engagement in therapeutic and community services in Black families compared to White families. Given the significant history and perpetuation of institutionalized racism in the medical field, satisfaction with the personal and emotional process of undergoing an Autism evaluation could be a much more salient factor for Black families regarding trust in providers’ conclusions and recommendations. Thus, it is important that providers be well trained in providing high-quality, culturally responsive care to promote continued engagement with clinical services, particularly in marginalized populations.
